# Dataset of inertial measurements for writing Punjabi characters using IMU sensors

**DOI:** 10.1016/j.dib.2024.111083

**Published:** 2024-11-08

**Authors:** AnchalPreet Sharma, Harsh Kumar, Lakhjeet Kaur, Ramakant Kumar, Pravin Kumar

**Affiliations:** a Department of Computer Science, Akal University, Bathinda, Punjab 151302, India; b Department of Computer Science, GLA University, Mathura, UP 281406, India

**Keywords:** Writing style diversity, Inertial measurement units, Handwritten alphabets, Sensors, Punjabi, Pattern recognition

## Abstract

This study introduces a comprehensive methodology for gathering datasets to recognize handwritten Punjabi alphabets, utilizing Inertial Measurement Units (IMUs) to capture the dynamic movement patterns inherent in handwriting. The approach considers the diverse writing styles found across Punjabi writers, which presents unique challenges due to regional variations in script. The dataset and collection system are designed to enhance recognition accuracy by harnessing this diversity.

The data collection process involved recording handwriting movements from multiple participants, ensuring the dataset reflects a wide range of writing styles. By leveraging IMUs, the system tracks detailed handwriting motions, enhancing character recognition accuracy.

The use of IMUs allows for the detailed tracking of handwriting movements, which is crucial for improving the accuracy of character recognition. Preliminary experimental results indicate that the dataset not only effectively captures the nuances of handwritten Punjabi but also demonstrates potential in recognizing handwritten English alphabets within the Indian context. This research contributes significantly to the field of pattern recognition, offering insights that could lead to the development of more robust handwriting recognition systems particularly suited for various linguistic and cultural settings.

Specification Table

**Table:** 

Subject	Data Science; Internet of Things data in linguistics; Pattern recognition in Punjabi character;
Specific Subject Area	Punjabi Characters Pattern Recognition
Type of data	CSV files
Data collection	An ESP32/ESP8266 development board, well-regarded for its compact design and compatibility with the Arduino platform, was used with an IMU 6050 sensor for data collection [1]. This setup was integrated into a marker pen to capture the inertial data associated with the writing of 41 Punjabi characters. Initially, the IMU 6050 sensor, capable of measuring acceleration and angular velocity, was mounted on the top of the marker. The sensor's placement at the top enabled the collection of detailed inertial data as each of the 41 Punjabi characters was written. This data provided insights into the pen's motion dynamics, including tilt, acceleration, and angular momentum during the writing process. Subsequently, the IMU 6050 sensor was repositioned at the bottom of the marker to observe any variations in inertial data that might occur due to the change in sensor placement. By collecting data from both positions, a comparative analysis could be conducted to understand how the location of the sensor affects the captured motion parameters. This dual-position approach ensures a comprehensive understanding of the writing dynamics and the influence of sensor positioning on data accuracy and interpretation. The collected data is intended to contribute to the development of handwriting recognition systems or to enhance the precision of motion-tracking applications.
Description of data collection	A system was developed for data collection using the ESP8266 microcontroller and the MPU6050 sensor. The MPU6050 sensor, which integrates both an accelerometer and a gyroscope, is responsible for capturing the movement and orientation of the smart pen. The ESP8266, a microcontroller with built-in Wi-Fi capability, facilitates the wireless transmission of this sensor data. **System Architecture:** **ESP8266 (Server):** The ESP8266 operates as the server, collecting sensor data from the MPU6050 and publishing it to a network. **MQTT Broker:** The MQTT broker [2] serves as the intermediary that manages the communication between the ESP8266 server and the client. It receives the data published by the ESP8266 and makes it available for subscribers. **Client (Laptop):** The laptop functions as the client in this setup, subscribing to the MQTT broker to receive the sensor data transmitted by the ESP8266 server.
	This architecture enables real-time data transmission and collection, with the ESP8266 publishing the sensor data to the MQTT broker, and the laptop client retrieving and processing the data as it is received.
Data source location	: https://data.mendeley.com/datasets/jpzz4gch7z/1 Akal University Talwandi Sabo,Bathinda
Data accessibility	Repository name: Mendeley Data [3] Data identification number: DOI: 10.17632/jpzz4gch7z.1 Direct URL to data: https://data.mendeley.com/datasets/jpzz4gch7z/1

## 1 Value of the Data

• *Education:* The character recognition dataset is used in educational applications to help students learn to read and write and to develop language skills [4]. For example, handwriting recognition software can help students practice writing letters and words [5]. The student writes a letter on a surface using a smart pen equipped with sensors that track its motion in two-dimensional space. The accelerometers measure linear movement along the x, y, and z axes (ax1, ax2, ax3), while the gyroscopes measure rotational movement around the x, y, and z axes (gx1, gx2, gx3) [6]. As the student writes, the smart pen streams this sensor data in real-time to a connected application on a tablet, smartphone, or computer via Bluetooth or another wireless method.
• *Handwriting analysis:* This dataset is used for handwriting analysis and studies handwriting to learn about the writer or check if documents are genuine. This technique is helpful in forensics to match handwriting to people, confirm signatures, and spot forgeries. Even without special marks called diacritics, the dataset can help identify different handwriting styles by looking at letter shapes, spacing, pressure, and line quality. Advanced technology and pattern recognition make this process even more accurate, helping to identify unique handwriting styles and verify documents.
• *Accessibility:* Character recognition datasets are crucial for creating tools that help people with disabilities. Dataset, which contains Punjabi characters without diacritics, is used to develop assistive technologies. These datasets train Optical Character Recognition (OCR) systems to scan printed or handwritten text and convert it into machine-encoded text [7]. For people who are blind or have low vision, OCR can be a powerful tool. It turns text from physical documents into digital text, which can be read aloud by text-to-speech (TTS) systems. These tools support screen readers, Braille devices, and other accessibility features on gadgets, making it easier for users to access books, educational materials, and signs.
• *Search Engines:* Using a Punjabi text dataset without diacritics, you can build search engines that match what users are looking for with the content. This helps users find the right documents, articles, or web pages even if the text doesn't have diacritical marks. It makes searches effective by focusing on the key terms or phrases, ensuring users can still find what they need.

## 2 Background

The main goal of the dataset is to use the MPU6050 Inertial Measurement Unit (IMU) sensor to fully record and analyze the motion data involved in producing Punjabi alphabets. This dataset intends to offer a solid basis for Punjabi letter detection and prediction by studying the dynamic motion patterns associated with handwriting. This will help to develop natural language processing and handwriting recognition technology.

This dataset focuses on the peculiarities of writing in Punjabi, which is more difficult than writing in English because of the script's complex curves and strokes. Six degrees of freedom are recorded by the MPU6050 sensor, three from the accelerometer and three from the gyroscope, giving precise motion information for every letter of the alphabet. In order to create machine learning models that can correctly recognize handwritten Punjabi characters, a thorough collection of data is essential.

To ensure a wide range of writing styles and speeds, the dataset collection process entails gathering data from a diverse group of participants, improving the final models' robustness and generalizability. This datasetʼs analysis attempts to answer essential concerns like the variation in handwriting among users and the effects of different sensor locations and environmental factors on data accuracy.

In the end, this dataset is a valuable tool for scientists and programmers who are developing handwriting recognition software, especially for Punjabi scripts. Additionally, it offers insights into the use of IMU sensors for the capture of intricate human motion patterns, supporting more significant initiatives in the fields of machine learning and IoT-based data collection.

## 3 Data Description

**Fig. 1:**
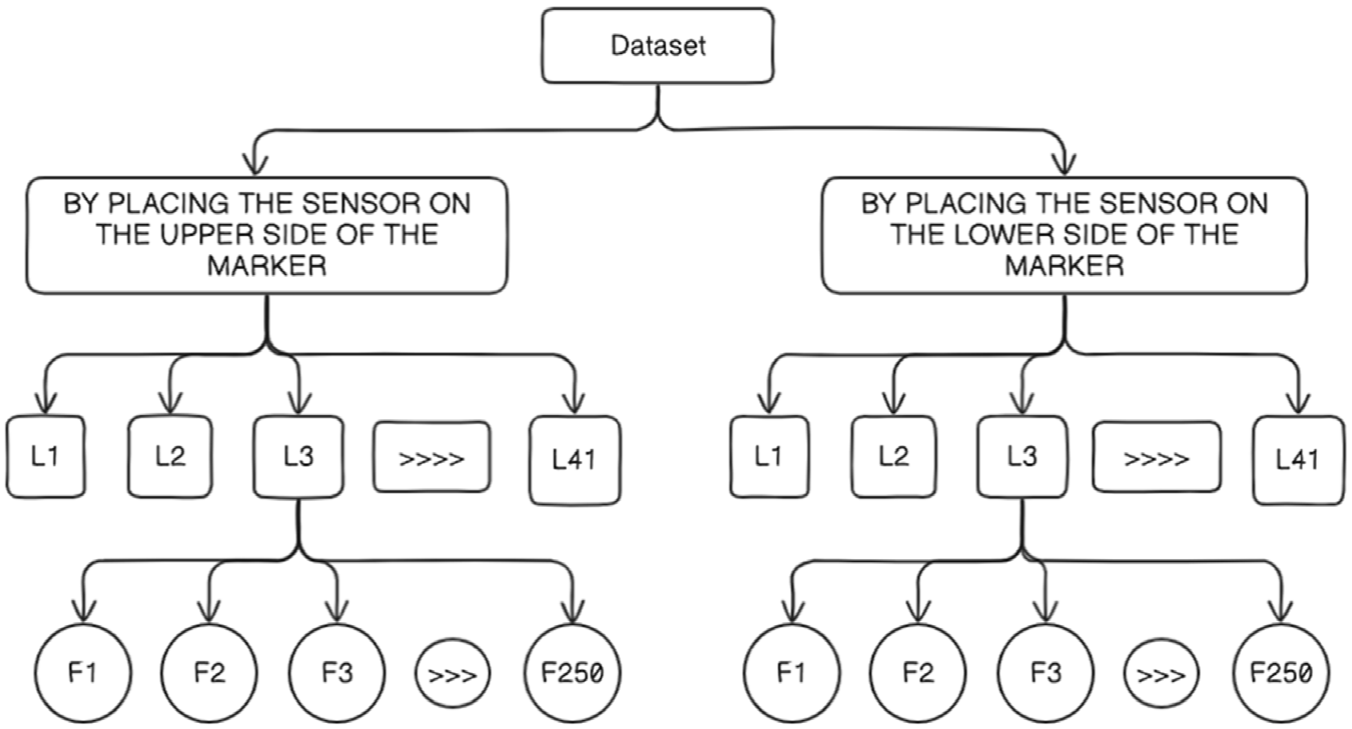
Overview of the dataset generated from 20 students writing the 82 letters of the Punjabi alphabet, with sensor data captured from both upper and lower marker positions.

**Fig. 2:**
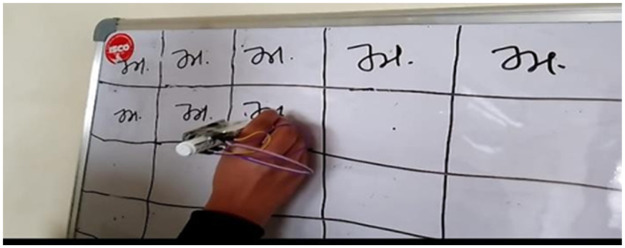
Example of characters being written on a whiteboard during a data collection session with the smart pen equipped with an ESP8266 and MPU6050 sensor.

**Fig. 3:**
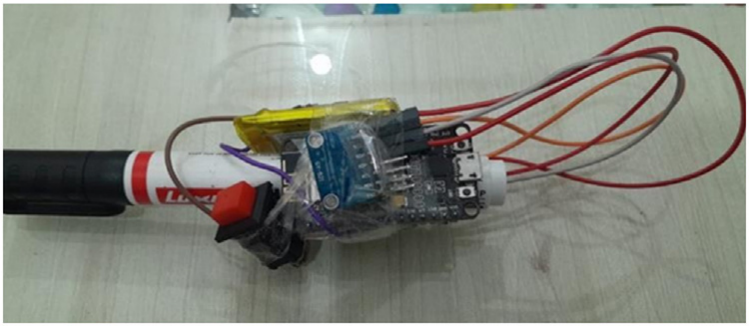
Experimental setup for data collection, where students wrote each character 250 times, using a button to record the timing and duration of each writing instance.

The data collection process involved the participation of 20 students who were assigned to write 82 letters of the Punjabi alphabet. This included 41 letters with the sensor attached to the upper part of the marker and 41 letters with the sensor attached to the lower part of the marker. The dataset created by this process has been illustrated in Fig. 1. The data is labeled by assigning the first character as 1, the second character as 2, and so on. The data collection experiment was conducted over a period of four months, ensuring a substantial amount of data was gathered. During each session, they wrote characters on the whiteboard, as shown in Fig. 2. The students were instructed to write each character 250 times on the board. A button was provided to the students to capture the timing and duration of each writing instance. They would press the button before starting to write a character, and after completing the character, they would release the button. The setup is shown in Fig. 3 the dataset collection, the sensor recorded data from all 250 instances of each character being written by the students. This comprehensive data collection approach enabled the capture of multiple repetitions of each character, providing a rich dataset for analysis and modeling purposes. The collected data offers valuable insights into the writing behavior and patterns exhibited by the students.

In our experiment, we also aim to understand how the position of the sensor affects acceleration and gyroscope readings while writing characters. To achieve this, we conducted data collection by placing the sensor at two distinct positions on the writing instrument: one at the end side of the marker and the other at the front side (near the nib). By doing so, we are able to capture how the sensor's location influences the dynamics of writing motions, which is crucial for analyzing the variations in acceleration and angular velocity across different points of the marker.

To thoroughly address the impact of sensor placement on data quality, itʼs essential to analyze and quantify how different placements (end vs. front side of the marker) affect the key metrics. The goal is to understand whether sensor placement introduces any significant changes in the captured data, influencing the motion patterns' accuracy, noise level, and consistency during character writing.

**The data is impacted in the following ways by the positioning of sensors on either side of the marker (end or close to the nib):**

1. *Sensor Placement:* This is closely related to the sensorʼs ability to record accurate movement data, whether the sensors are placed on the front side (near the tip) or the markerʼs end. According to the study, precise hand movement dynamics detection depends on the placement of the sensor.
2. *Impact of Placement:* The characteristics of the data are altered by the sensorʼs position. For instance, a sensor at the end (back) records wider motions associated with hand positioning and arm movement, while a sensor near the nib (front) records finer, more specific movements involved in making letters. Variations in gyroscope and accelerometer readings, which represent rotation and acceleration along separate axes, may result from this distinction.
3. *Gyroscope Sensitivity:* The sensitivity of the gyroscope varies with the height of the writer and the angle of the marker, especially around the y-axis. Larger rotational arcs might be tracked by a sensor at the back, while minor motions could be detected by a sensor near the nib (front side).
4. *Overall Influence on Data:* We claim that sensor placement affects how well MPU605 captures writing dynamics, including rotating speed and writing angle. We notice distinct motion patterns by contrasting the sensors at the rear and near the tip, which gives us richer data for handwriting recognition that is more accurate.

This analysis highlights how the systemʼs accuracy for handwriting recognition tasks is improved by selecting the right sensor location on the marker.

To ensure a comprehensive dataset that reflects a wide range of writing styles and movements, we carefully selected volunteers with varying levels of proficiency in Punjabi. This group included individuals who were highly fluent in Punjabi, as well as those with less fluency. By incorporating participants with different levels of language proficiency, we aimed to capture diverse motor patterns and handwriting nuances. This approach allows for a more robust analysis of the inertial data, as it encompasses the variations in movement that occur due to differences in familiarity with the language and writing practice.

**Fig. 4:**
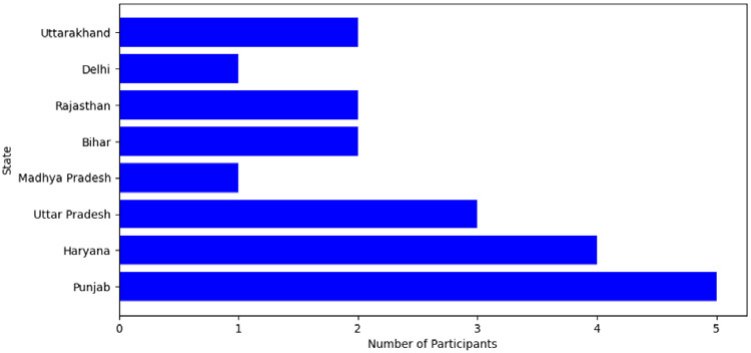
Illustration of volunteer distribution in terms of geographic regions in India.

The students were selected from various geographical regions across India. Table 1 and Fig. 4 provides a breakdown of the number of participants from each region.

**Table 1:** Distribution of participants from different geographical regions of India.

State	No. of participant
Punjab	5
Haryana	4
Uttar Pradesh	3
Madhya Pradesh	1
Bihar	2
Rajasthan	2
Delhi	1
Uttarakhand	2

### 3.1 Participant selection and generalizability

In this study, participants were selected from various regions and states. However, since the primary focus was on analyzing sensor data (acceleration and gyroscope readings) collected during character writing, the geographic origin of the participants is not expected to influence the results. Handwriting movement patterns, as measured by the sensors, are largely shaped by motor skills rather than regional or cultural variations.

To ensure consistency, participants were selected based on a similar level of writing proficiency, allowing the study to generalize findings about sensor placement on the marker (end vs. front) and its impact on data quality during character writing. The diverse backgrounds of the participants enhance the robustness of the results and help confirm that they are not biased by geographic or cultural factors.

A limitation of this study is that all participants were right-handed. In future research, we plan to include left-handed individuals to expand the scope of the analysis.

All participants had a similar level of writing proficiency, minimizing variability in writing style.

**Fig. 5:**
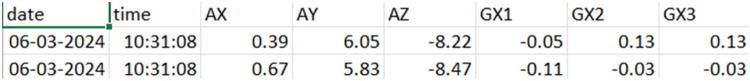
Example of the six-axis sensor data, including three axes of accelerometer readings and three axes of gyroscope readings.

The data collected during the process consists of six-axis measurements, including three axes from the accelerometer and three axes from the gyroscope, as illustrated in Fig. 5.

The following image presents a snippet of the dataset, with columns corresponding to various sensor readings. Here's a breakdown of the columns:

1. **Date**: This column contains the date on which the data was recorded (in this case, “06-03-2024”).
2. **Time**: This column contains the exact time the data was recorded (in this case, “10:31:08”).
3. **AX, AY, AZ**: These columns represent the accelerometer readings along the X, Y, and Z axes, respectively. Accelerometers measure the acceleration force in these directions.
4. **GX1, GX2, GX3**: These columns represent the gyroscope readings along the X, Y, and Z axes, respectively. Gyroscopes measure the rate of rotation around these axes.

During the data collection process, a specific protocol was followed to organize and record the data. Each file in the dataset represents a particular character, and within that file, the participant wrote the same character 250 times. All 250 instances of writing that character by a participant were recorded in this file. To initiate the recording of each writing instance, the student pressed a button. Along with these values, readings from sensors such as accelerometers, gyroscopes, and were also captured.

This ensured that there was a separate file for every instance of writing the character. This allowed for further analysis and examination of patterns, variations, and trends within and across participants. Additionally, having individual files for each instance and participant facilitated the isolation and study of specific writing behaviors, helping to identify unique characteristics or writing a particular character. Overall, this approach ensured that the data collected was structured and categorized to facilitate subsequent analysis and modelling tasks.

**Fig. 6:**
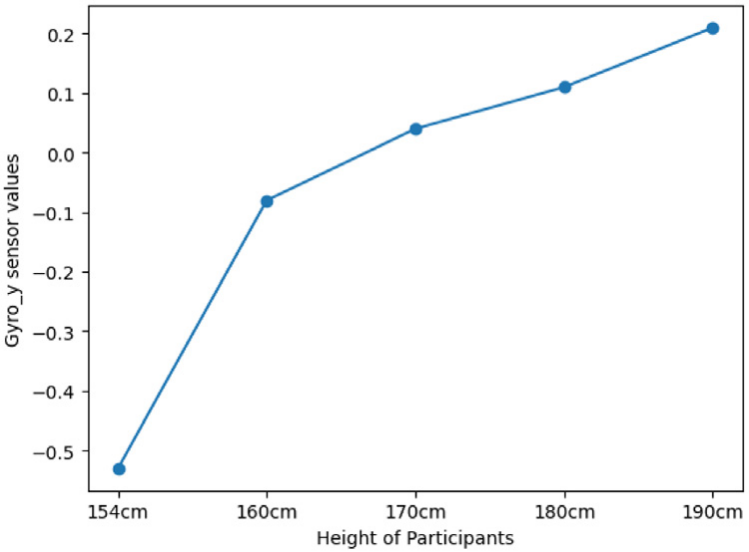
Relationship between height and gyro Y-axis sensor values, showing increased angular velocity with greater height during writing on the whiteboard.

We observed that the gyro Y-axis sensor values increase with height, indicating that taller individuals exhibit higher angular velocity around the Y-axis compared to shorter individuals while writing on the whiteboard. This trend is depicted in Fig. 6.

**Fig. 7:**
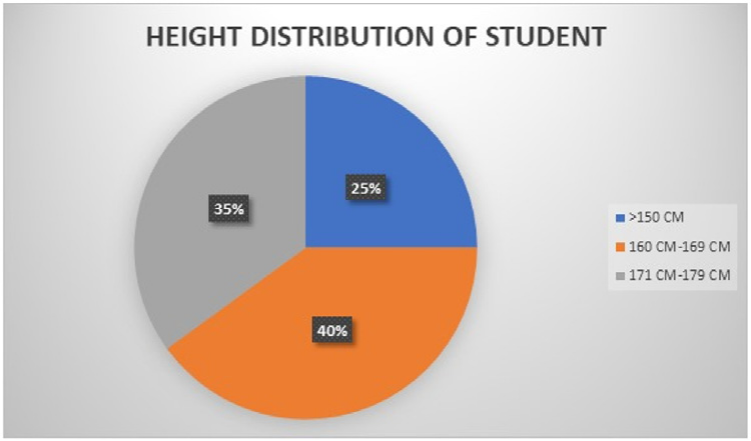
Height distribution of student volunteers who participated in the data collection for the handwritten Punjabi alphabet dataset using the MPU6050 sensor.

The student volunteers varied in height and gender, contributing to the diversity of the dataset. Fig. 7 illustrates the height distribution of these volunteers, who were involved in collecting the handwritten Punjabi alphabet characters dataset using the MPU6050 sensor.

**Fig. 8:**
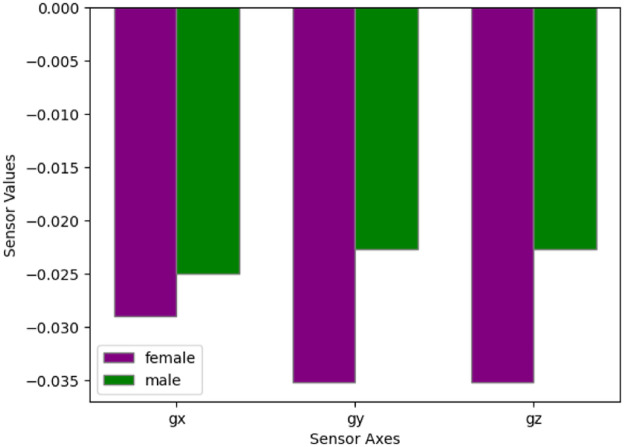
Comparison of accelerometer values between male and female volunteers, highlighting the impact of gender on sensor measurements.

**Fig. 9:**
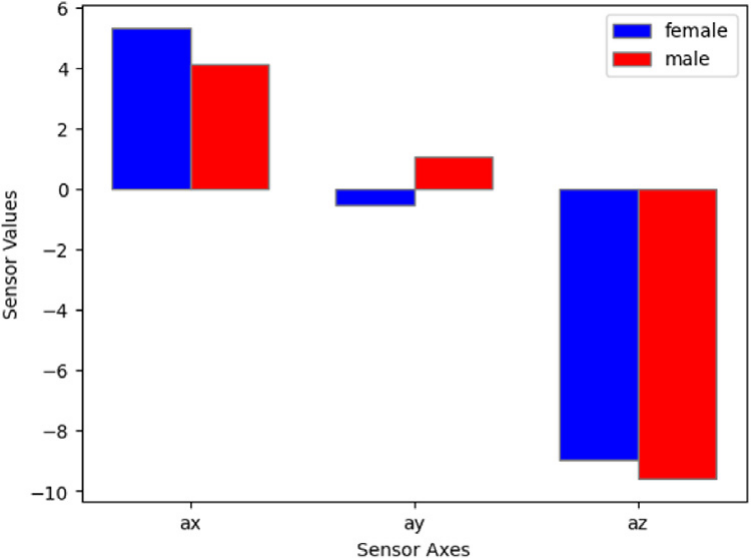
Comparison of gyroscope values between male and female volunteers, highlighting the impact of gender on sensor measurements.

An illustrate of the impact of male vs female volunteers on the values of the accelerometer, and gyroscope as shown in Fig. 8, Fig. 9.

## 4 Experimental Design, Materials and Methods

### 4.1 Dataset composition

To assemble a dataset for an IoT-based application using an ESP8266 Wi-Fi module, MPU6050 sensor, battery system, and momentary switch, consider the following key components:

**ESP8266:** A low-cost system-on-chip (SoC) was introduced in 2014, featuring a TCP/IP protocol stack that enables microcontrollers to connect to Wi-Fi networks, making it ideal for IoT applications.

**MPU6050 Sensor:** Provides three-axis accelerometer and gyroscope data, crucial for detecting motion and tracking orientation.

*Battery System:* Ensures portability and autonomy by providing stable, efficient, and potentially rechargeable power to the ESP8266 and MPU6050 modules.

*Momentary Switch:* This enables transient electrical connections, essential for initiating data transmission between the MPU6050 sensor and the ESP8266 module.

**Fig. 10:**
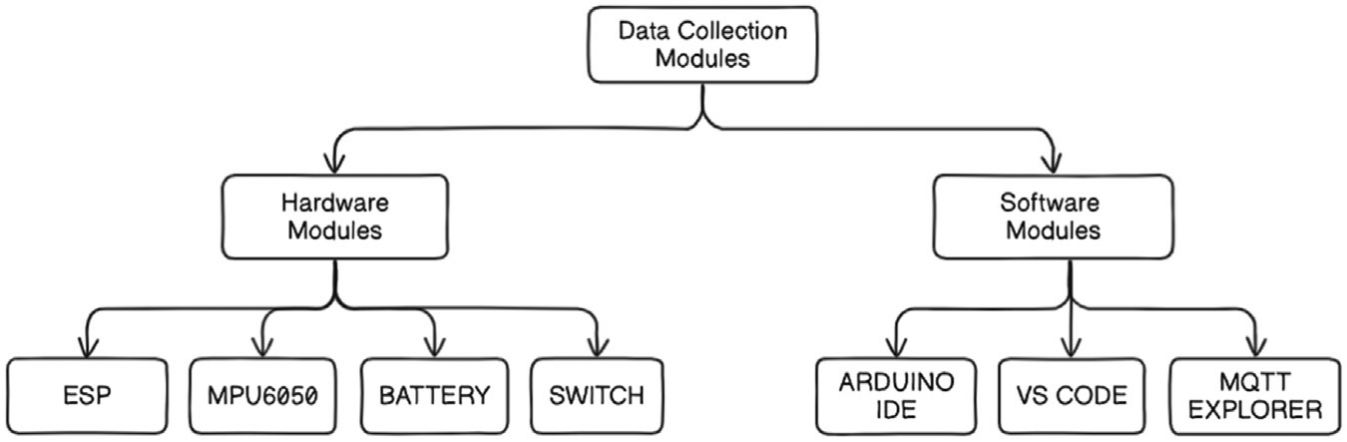
Overview of the hardware and software components required for data collection in an IoT-based application using the ESP8266, MPU6050 sensor, battery system, and momentary switch.

Fig. 10 illustrates the hardware and software components required for data collection in this IoT setup. Table 2 shows the material required for the data collection. Table 3 lists the materials required for the data collection process, including all essential components and their specifications necessary for assembling the IoT-based data collection system.

**Table 2:** Summary of participant selection and sensor setup.

Category	Description
Number of Participants	20
Regions/States Represented	Multiple (Participants from different states/regions)
Impact of Geographic Origin	No significant impact on sensor readings (acceleration and gyroscope)
Writing Task	Writing 82 Punjabi characters (41 with the sensor placed on end side of the marker, 41 with the sensor placed on the front side of the marker(near the tip))
Sensor Placement	Two configurations: End side of the marker and front side (near the tip) of the marker
Data Collected per Character	250 samples per character for both end and front side of sensor placements
Measurement Focus	Acceleration and gyroscope readings during character writing
Purpose of Study	Investigating the impact of sensor placement on data quality during handwriting recognition experiments
Participant Handwriting Skill	All participants had a similar level of writing proficiency, minimizing variability in writing style

**Table 3:** List of materials required for the data collection process, including specifications for each component used in the IoT-based system.

Sr.No	Component	Quantity
1.	Arduino Uno	1
2.	VS Code	1
3.	ESP8266	1
4.	MPU6050	1
5.	Switch	1
6.	Battery	1
7.	Jumper wire	6
8.	Marker	1
9.	Black-Board	1

### 4.2 Experimental setup and design

#### 4.2.1 Sensors and devices

The MPU6050 is an Inertial Measurement Unit (IMU) sensor that integrates a single chip's 3-axis accelerometer and a 3-axis gyroscope.

**Accelerometer:** Measures acceleration along the X, Y, and Z axes. This can be used to detect changes in velocity, orientation, and the force of gravity.

**Gyroscope:** Measures rotational speed around the X, Y, and Z axes. It helps detect angular velocity, which is useful for tracking orientation changes.

**Steps to Capture Data:**

1. *Initialization:* The MPU6050 sensor is powered up and initialized by configuring its registers, typically via I2C communication. The microcontroller sends configuration data to the sensor to set it up in the required mode.
2. *Reading Data:* The sensor continuously captures raw data from the accelerometer and gyroscope and stores it in its internal registers.
3. *Data Retrieval:* The microcontroller (e.g., ESP8266) reads the raw data at specific intervals. This data typically consists of values for each axis of the accelerometer and gyroscope.

**Fig. 11:**
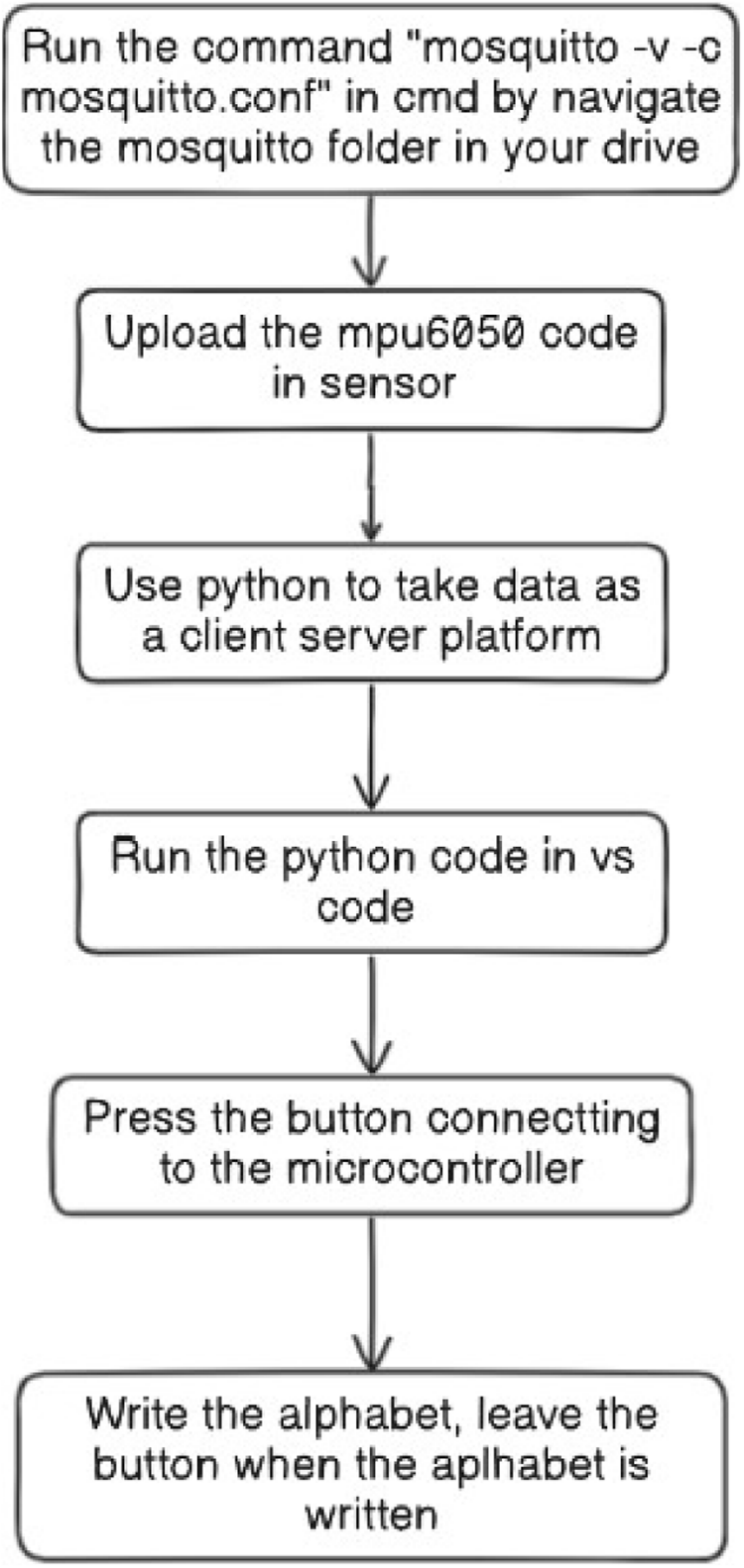
Flowchart depicting the process of making a dataset.

The step to capture has been shown in Fig. 11.

#### 4.2.2 ESP8266 microcontroller

The ESP8266 is a low-cost Wi-Fi microcontroller with a built-in TCP/IP stack and capabilities for handling I2C communication with sensors like the MPU6050.

Steps Involved:

1. *Connection Setup:* The ESP8266 is connected to the MPU6050 via the I2C bus (SCL and SDA lines). The sensor's power (VCC) and ground (GND) are connected to the respective pins on the ESP8266.
2. *Data Processing:* The ESP8266 retrieves raw accelerometer and gyroscope data from the MPU6050. This data may need to be processed or filtered (e.g., using a complementary filter) to obtain meaningful information such as angle or displacement.
3. *Wireless Communication Setup:* The ESP8266 is connected to a Wi-Fi network to enable wireless data transmission.

### 4.3 Wireless data transfer using MQTT protocol

MQTT (Message Queuing Telemetry Transport) is a lightweight messaging protocol well-suited for IoT applications, facilitating the wireless transfer of sensor data

Steps for Wireless Data Transfer:

MQTT Client Setup: The ESP8266 is configured as an MQTT [8] client using a library such as PubSubClient. It establishes a connection to an MQTT broker— a server that routes messages between clients—over the Wi-Fi network.

Data Formatting: Sensor data is formatted into CSV before transmission. This standardizes the data for easier parsing and processing by the receiving system.

Publishing Data: The ESP8266 publishes the formatted sensor data to a designated MQTT topic, which acts as a communication channel. The MQTT broker then forwards this data to any devices or applications subscribed to the topic.

Subscription and Retrieval: Devices or applications subscribed to the topic can receive and process the data in real time. For instance, a PC or cloud service can subscribe to the topic to display or store the incoming data.

Sensor Placement: The sensor placed on the top of the marker captures the movement and orientation of the upper part of the marker.

• *Participants and Data Collection Protocol:* The participants' demographics, the way in which they interacted with the setup, and the procedure for starting and stopping each data recording session by pressing a button.

Materials

• **Components Used**:
  ○ **ESP8266**: For wireless data transmission.
  ○ **MPU6050**: For capturing motion and orientation data.
  ○ **Arduino Uno**: As the main microcontroller board.
  ○ **Switch**: To initiate data recording.
  ○ **Battery**: Power source.
  ○ **Marker and Black-Board**: Used in the writing experiment.
• **Software**:
  ○ **VS Code**: Mention its use as the integrated development environment (IDE) for programming the ESP8266.
  ○ **MQTT Broker**: This is for managing communication between devices.

### 4.4 Comparative studies between dataset of inertial measurements for writing Punjabi characters and other existing work

**Sensor Technology**

*Our Study:* The MPU6050 sensor, a 6-axis Inertial Measurement Unit (IMU), was utilized in this study to record hand dynamics motion data. With the use of this sensor, which has a 3-axis accelerometer and gyroscope, writing-related hand movements can be measured in both fine and wide ways. Sensors were carefully positioned on the front (next to the nib) and rear of the marker to improve data collecting.

*Existing Study:* The LSM9DS1 and MPU9250 sensors, which are 9-axis IMUs with a magnetometer built in, were used in the cited work. In comparison to the MPU6050, this addition improves the overall accuracy of motion analysis by offering more orientation and magnetic field strength data, enabling more thorough movement tracking [9].

**Data Collection Setup**

*Our Study:* The MPU6050 sensor was used to gather data, and it was positioned at the front and back of the marker. It recorded motion data at a 50 Hz sample rate. The combination of these two sensors allowed for the precise recording of both small and large hand movements when writing Punjabi characters.

*Existing Study:* An Arduino Nano BLE Sense with LSM9DS1 sensors placed at the tip and upper end of the marker was used in the comparison study. This configuration was intended to record both big hand movements and minute wrist movements close to the marker's nib [9].

**Alphabet Type and Writing Styles**

*Our Study:* With their complex and continuous stroke patterns, the 41 Punjabi alphabets in the Gurmukhi script are the subject of this dataset. This writing style's inherent intricacy incorporates curved and looping movements, which creates special difficulties for motion data recording.

*Existing Study:* 52 English alphabets (26 capital and 26 lowercase), which often have more recognizable letter forms and simpler strokes, were studied in the current study. English letter handwriting is more distinct and non-continuous than other handwriting styles, which leads to simpler hand motion patterns [9].

**Dataset Size and Structure**

*Our Study:* Each alphabet has 250 files in the dataset, which are all captured for front and back sensor positions. This yields 20,500 files per side (41 alphabets total), which provides a strong and complete set of motion data for analysis, with each file representing a single alphabet instance.

*Existing Study:* Each of the 124 participants in the comparative study contributed 50 occurrences of both capital and lowercase letters to the dataset. This comprehensive gathering produced a sizable dataset with numerous iterations of every letter, which was helpful for challenges involving the recognition of handwriting in general. Table 4 list the comparative studies between the English alphabet and the Punjabi alphabet [9].

**Table 4:** Comparative analyses between our work and existing work.

Feature	Our Study (Punjabi Alphabets)	Referenced Study (English Alphabets) [9]
**Sensor Technology**	MPU6050 (6-axis: accelerometer, gyroscope)	LSM9DS1 (9-axis: accelerometer, gyroscope, magnetometer), MPU9250
**Sensor Placement**	Front (near nib) and back of the marker	Tip of the marker and upper end
**Sampling Rate**	50 Hz	50 Hz
**Alphabet Type**	41 Punjabi alphabets (Gurmukhi script)	26 uppercase and 26 lowercase English alphabets
**Writing Style**	Complex curved strokes, continuous flow, distinct letter dynamics	Simpler, less curved, distinct upper and lower case letters
**Dataset Size**	41 alphabets × 250 files per sensor placement (20,500 files total)	52 alphabets × 50 repetitions × 124 participants (Large dataset)
**File Structure**	Each file captures the motion sequence for a single letter per side	Each file captures multiple repetitions of a single letter
**Handwriting Dynamics**	Continuous hand movements, complex trajectories	Simpler, more discrete strokes and curves
Applications	Punjabi script recognition, regional NLP tools, educational aids	English script recognition, handwriting analysis, sign language

## Limitation

*Sensor Accuracy and Calibration:* The MPU6050 sensor's accuracy can be influenced by calibration errors. Inaccurate calibration may lead to bad data, affecting the quality of the collected motion and orientation measurements.

*Wireless Communication Reliability:* The performance of the ESP8266 and MQTT protocol depends on Wi-Fi signal strength and network stability. Interference like network congestion or signal loss can result in data transmission delays or loss [9].

*Limited Sensor Range:* The MPU6050 sensor has specific ranges for acceleration and angular velocity. If the writing motions exceed these ranges, the sensor may not capture the full extent of the movements, leading to incomplete data.

*Left-Handed Individual Dataset:* The current dataset was created by right-handed individuals, which is a limitation. In the future, we plan to create a dedicated dataset from left-handed participants and submit it to the databank. Additionally, we are planning a comparative data analysis between right- and left-handed individuals to explore potential differences.

## Sensitivity of MPU6050

The MPU6050 accelerometer supports a user-programmable full-scale range of ±2g, ±4g, ±8g, and ±16g, which allows for flexibility depending on the desired sensitivity. In your case, using ±4g is effective for capturing detailed and subtle movements. Similarly, the gyroscope's range is programmable to ±250, ±500, ±1000, or ±2000° per second (DPS), making ±500 dps suitable for accurately tracking rotational motion. https://cdn-learn.adafruit.com/downloads/pdf/mpu6050-6-axis-accelerometer-and-gyro.pdf.

## Data Availability

Mendeley DataA Dataset of Inertial Measurement Units for Handwritten Punjabi Alphabets.
